# When cancer reaches the heart: a rare presentation of cardiac metastasis with atrioventricular block and left ventricular outflow tract obstruction

**DOI:** 10.1093/ehjcr/ytae654

**Published:** 2024-12-07

**Authors:** Çetin Alak, Dilek Yeşilbursa, Bülent Özdemir

**Affiliations:** Department of Cardiology, Uludag University School of Medicine, Bursa 16059, Turkey; Department of Cardiology, Uludag University School of Medicine, Bursa 16059, Turkey; Department of Cardiology, Uludag University School of Medicine, Bursa 16059, Turkey

## Summary

Metastatic involvement of the heart, although less frequent than primary cardiac tumours, poses a significant complication of malignancy. Secondary tumours often lead to pericardial effusion rather than direct myocardial infiltration. Common cancers with cardiac metastasis include melanoma and primary mediastinal tumours, which can cause obstructive lesions and arrhythmias.^1^ The occurrence of complete heart block due to metastatic disease is particularly rare.

**Figure 1:**
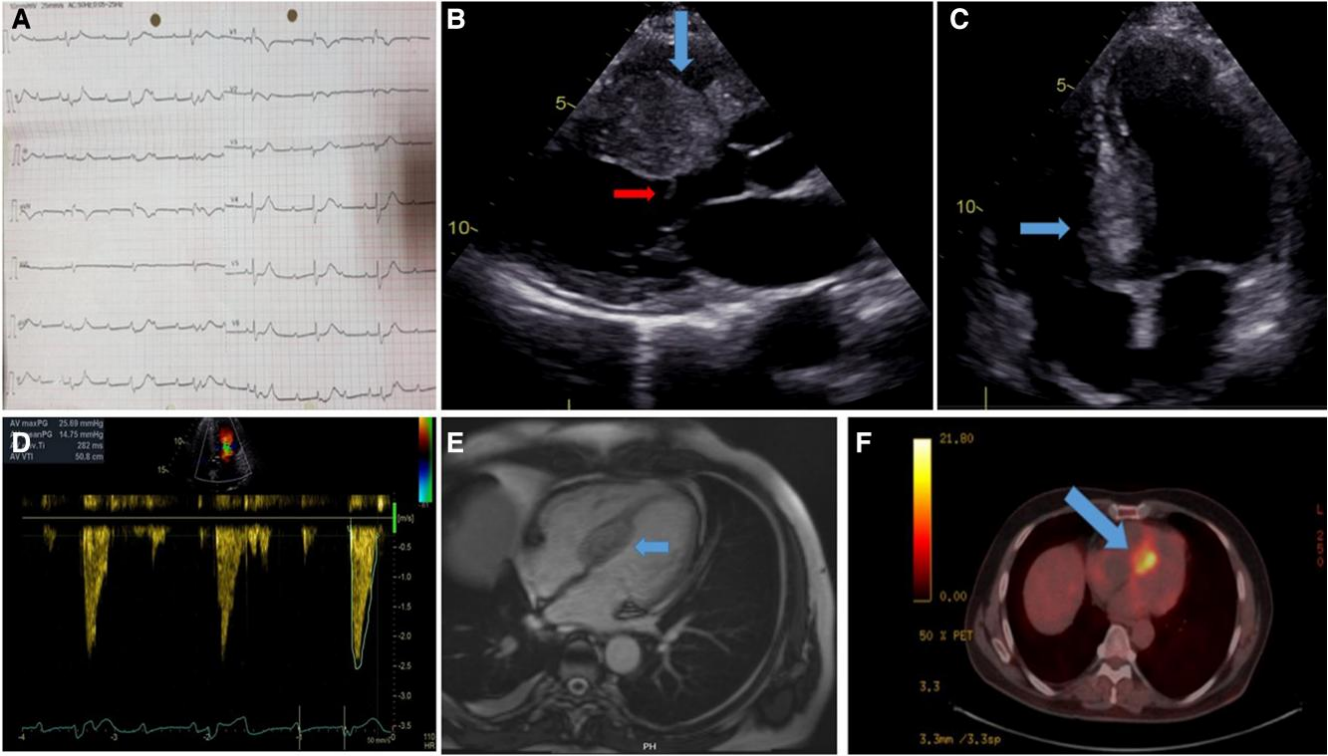
Multiple-panel image illustrating: (*A*) electrocardiogram (EKG) illustrating the presence of complete atrioventricular (AV) block, characterized by the dissociation between atrial and ventricular rhythms. (*B*) Transthoracic echocardiography in the parasternal long-axis view, with the blue arrow indicating interventricular septal hypertrophy and the red arrow highlighting the subvalvular membrane. (*C*) Transthoracic echocardiography in the four-chamber view, with the blue arrow indicating prominent interventricular septal hypertrophy. (*D*) Continuous Doppler wave image demonstrating an obstruction in the subaortic area with a maximum gradient of 25 mmHg, a velocity of 2.5 m/s, and a dagger sign. (*E*) Cardiac MRI balanced steady-state free precession (SSFP) cine frame, with the blue arrow indicating a mass within the interventricular septum, suggestive of metastatic infiltration. (*F*) Positron emission tomography-computed tomography scan with the blue arrow indicating increased metabolic activity in the interventricular septum, consistent with the presence of the mass.

This report describes a 64-year-old male with a history of lung adenocarcinoma presented with fatigue and worsening dyspnoea. Initial evaluation revealed a third-degree atrioventricular (AV) block, significant septal hypertrophy (28 mm), and subaortic obstruction with a maximum gradient of 25 mmHg. Advanced imaging with cardiac magnetic resonance imaging (MRI) and positron emission tomography-computed tomography (PET-CT) identified a mass encircling both ventricular outflow tracts and increased metabolic activity in the interventricular septum, consistent with metastasis (*Figure 1*).

We discuss the pathophysiological mechanisms through which cardiac metastasis can cause both AV block and left ventricular outflow tract (LVOT) obstruction. Direct myocardial invasion by metastatic tissue disrupts the normal conduction pathways, leading to AV block, while mass effect within the septum can lead to LVOT obstruction by physically impinging on the outflow tract and altering hemodynamics.^2^

This case is notable for the rare occurrence of cardiac metastasis resulting in both complete AV block and LVOT obstruction—previously reported only once in the literature, and that diagnosis was established post-mortem.^2^ The ability to diagnose such conditions pre-mortem through multimodality imaging underscores the essential role of advanced imaging in identifying atypical manifestations of metastatic cancer.

## Case description

A 64-year-old male with a history of lung adenocarcinoma presented with fatigue and worsening shortness of breath. On examination, his heart rate was 45 beats per minute with third-degree AV block noted on electrocardiogram, blood pressure was 114/76 mmHg, and a 3/6 systolic murmur was audible at the aortic focus. Transthoracic echocardiography showed significant septal hypertrophy (28 mm) with subaortic obstruction, yielding a peak gradient of 25 mmHg (*Figure 1*). Additionally, a subvalvular membrane was identified, suspected to be related to metastatic infiltration rather than a pre-existing condition given the patient’s oncologic history and new myocardial findings. Although the Valsalva manoeuvre is frequently employed to assess dynamic LVOT obstruction, it was not performed in this patient due to haemodynamic concerns and potential instability.

Advanced imaging with cardiac MRI revealed a mass extending from the interventricular septum into the inter-arterial space, suggestive of metastatic tissue infiltration rather than a primary cardiac neoplasm. A balanced steady-state free precession (SSFP) cine frame on MRI highlighted the presence of the mass within the septum. Subsequent PET-CT demonstrated increased metabolic activity in the interventricular septum, consistent with metastasis.

Management initially focused on stabilizing the complete AV block, with a temporary pacemaker implanted and later replaced by a permanent dual-chamber DDD-R pacemaker. Due to the subaortic obstruction gradient being below 50 mmHg, conservative management was chosen for the LVOT obstruction. Supportive care, including rate control and symptom management, was provided, while surgical intervention was deemed inappropriate given the patient’s prognosis and overall metastatic disease burden.

Periodic imaging follow-ups showed no progression in septal hypertrophy or LVOT obstruction. However, despite stable cardiac status, the patient succumbed to septic shock seven months later.

## Supplementary Material

ytae654_Supplementary_Data

## Data Availability

No new data were generated or analysed in support of this case report. All relevant case details are incorporated within the article.
